# Mechanistic Insights Into the Controlled Release and Antioxidant Protection of *Neolamarckia cadamba* Bioactives in Probiotic Yogurt: A Molecular and Microstructural Approach

**DOI:** 10.1155/ijfo/8849423

**Published:** 2026-06-29

**Authors:** Sadhna Mishra, Arvind Kumar, Vinay Kumar, Ashutosh Rai, Manju Yadav, Vigya Mishra, Sunil Kumar Yadav, Dipendra Kumar Mahato

**Affiliations:** ^1^ Department of Dairy Science and Food Technology, Institute of Agricultural Sciences, Banaras Hindu University, Varanasi, Uttar Pradesh, India, bhu.ac.in; ^2^ Faculty of Agricultural Sciences, GLA University, Mathura, Uttar Pradesh, India, gla.ac.in; ^3^ Department of Genetics and Plant Breeding, MS Swaminathan School of Agriculture, Centurion University of Technology and Management, Paralakhemundi, Odisha, India, cutm.ac.in; ^4^ Division of Vegetable Production, ICAR-Indian Institute of Vegetable Research, Varanasi, Uttar Pradesh, India; ^5^ Department of Home Sciences, Government P.G. College, Joshimath, Uttarakhand, India; ^6^ Department of Post Harvest Technology, College of Horticulture, Banda University of Agriculture and Technology, Banda, India; ^7^ ICAR-Indian Agricultural Statistics Research Institute, New Delhi, India; ^8^ CASS Food Research Centre, School of Exercise and Nutrition Sciences, Deakin University, Burwood, Victoria, Australia, deakin.edu.au

**Keywords:** encapsulation, *Neolamarckia cadamba*, phytochemical stability, probiotic yogurt, release kinetics

## Abstract

Fortification of functional dairy products with botanical bioactives is constrained by the inherent bitterness and chemical instability of plant extracts. This study investigated the microencapsulation of *Neolamarckia cadamba* (Kadam) bark extract for incorporation into probiotic yogurt, targeting preserved antioxidant activity and enhanced sensory acceptability. Ultrasonication‐assisted extraction in a 1:1 hydroethanolic system significantly outperformed conventional maceration in phytochemical recovery. The concentrated extract was spray‐dried (inlet: 160°C; outlet: 80°C) using an optimized *Quillaja* saponin–sunflower lecithin carrier matrix, yielding spherical, fracture‐free microcapsules with a principal intensity peak diameter of 78.98 nm (Z‐average: 6183 nm, indicative of polydisperse aggregate populations in the feed emulsion), and a highly stable zeta potential of −38.3 mV. SEM imaging confirmed successful tannin sequestration within intact particles. Functional evaluation of the fortified probiotic yogurt demonstrated a total phenolic content of 71.47 mg/g and DPPH radical scavenging activity of 72.94%, both sustained throughout refrigerated storage. A 42‐day release kinetics study confirmed controlled, gradual polyphenol diffusion into the yogurt matrix. Sensory panels awarded encapsulated formulations an overall acceptability score of 8.16 ± 0.36 out of 9, with no statistically significant differences observed between encapsulated and free‐extract treatments for any sensory attribute (all *p* > 0.05), indicating comparable sensory acceptability. These findings establish encapsulated *N. cadamba* extract as a physicochemically stable, clean‐label functional ingredient with improved antioxidant retention, favorable sensory acceptability comparable to free‐extract formulations, and controlled polyphenol release in a probiotic yogurt matrix. In vitro digestion and in vivo bioavailability studies are warranted to fully substantiate health‐efficacy claims.

## 1. Introduction

The integration of botanical bioactive compounds into fermented dairy products like yogurt represents a significant advancement in functional food development, driven by increasing consumer demand for prescriptive nutrition [1]. *Neolamarckia cadamba*, a tropical tree with traditional ethnomedicinal uses, is being explored for its potential in this area due to its rich secondary metabolite profile, including indole alkaloids such as cadambine and cinchonine, as well as polyphenols and tannins [2–5]. These phytochemicals have demonstrated potent antioxidant and antimicrobial properties, making *N. cadamba* an attractive candidate for yogurt fortification [6–9]. However, direct incorporation of *N. cadamba* bark extract into yogurt faces considerable challenges, primarily due to the intense astringency caused by tannins and alkaloids and the inherent instability of its bioactive compounds in the acidic yogurt matrix [10].

Yogurt, a widely consumed fermented dairy product, typically has a pH ranging from 4.0 to 4.6, which is beneficial for its preservation and the characteristic tangy flavor [11]. This acidic environment, although inhibiting less acid‐tolerant microbes, can lead to the degradation or undesirable cross‐linking of sensitive polyphenols with milk proteins, potentially causing syneresis or wheying‐off and compromising the product′s structural homogeneity during refrigerated storage [1, 12]. To overcome these challenges, encapsulation technologies are emerging as a strategic intervention for both flavor masking and the preservation of bioactivity [13]. Encapsulation creates a physical barrier around the bioactive compounds, shielding them from the acidic dairy environment, masking unpleasant flavors, and protecting them from degradation by light and oxygen (O) [14–16]. This approach also enables controlled release of the compounds, ensuring their stability throughout shelf life and their progressive release during consumption, without adversely affecting the delicate physicochemical and sensory attributes of the yogurt [17–19].

Studies have consistently highlighted the benefits of fortifying yogurt with various plant extracts to enhance its functional properties. For instance, the addition of *Moringa oleifera* leaf extract and *C. bonducella* seed extract has been shown to improve the physicochemical, microbiological, and antioxidant attributes of yogurt [20–23]. Similarly, extracts from *C. bonducella*, *Adiantum capillus-veneris* L., *Solanum melongena*, *Chrysanthemum*, ginger, blue butterfly pea, and ivy have all demonstrated potential in enhancing yogurt quality [20, 23–27]. The fortification of yogurt with functional ingredients boosts not only its nutritional value but also its biofunctional properties, including antioxidant, antidiabetic, and antihypertensive activities [21, 28, 29].

The process of fermenting dairy products, including yogurt, relies on the metabolic activity of lactic acid bacteria (LAB) such as *Lactobacillus* and *Streptococcus thermophilus* [11, 30, 31]. These microorganisms convert sugars, primarily lactose, into lactic acid, which lowers the pH of the milk, causing casein proteins to coagulate and form the characteristic yogurt texture. The stability of these probiotic cultures is crucial for the functional benefits of fortified yogurts. Encapsulation can further protect these sensitive cultures, along with the added plant bioactives, ensuring their viability and efficacy throughout storage [11, 32].

Research into *N. cadamba* has documented its bark, leaves, and fruits as rich sources of secondary metabolites, including strictosidine‐derived monoterpenoid indole alkaloids (MIAs) (cadambine and cinchonine), flavonoids (quercetin and gallic acid), tannins, and polysaccharides with demonstrated antioxidant, antidiabetic, wound‐healing, antileishmanial, antifungal, and antiplasmodial activities [2–7, 9, 33–35]. Despite this broad bioactivity profile, the systematic application of encapsulation technology to *N. cadamba* bark extract for incorporation into functional dairy products remains largely unexplored in the current peer‐reviewed literature.

However, the direct application of *N. cadamba* bark extract in yogurt fortification, specifically involving encapsulation and its impact on physicochemical and sensory properties, remains an area requiring extensive research. Although there is a strong rationale for using encapsulation to mitigate the challenges of astringency and instability associated with polyphenols and alkaloids in acidic dairy environments, the systematic evaluation of this specific application is largely unexplored in the current peer‐reviewed literature [13, 15]. Methods for characterizing successful encapsulation, such as Fourier transform infrared (FTIR) spectroscopy for chemical bonding and scanning electron microscopy (SEM) for morphological structure, are well‐established in food science. Similarly, the mathematical modeling of release kinetics is a standard approach to understand the controlled delivery of encapsulated compounds [16, 36, 37].

Against this background, the present study addresses the following objectives: (i) to compare agitation‐assisted extraction (AAE) and ultrasound‐assisted extraction (UAE) in aqueous and hydroethanolic solvents for recovery of antioxidant‐active phytochemicals from *N. cadamba* bark; (ii) to formulate and characterize spray‐dried *Quillaja* saponin–sunflower lecithin microcapsules using dynamic light scattering (DLS), zeta potential, SEM, FTIR, energy dispersive X‐ray (EDX), thermogravimetric analysis (TGA), and X‐ray diffraction (XRD); (iii) to evaluate the physicochemical, textural, and sensory attributes of probiotic yogurt fortified with free versus encapsulated extract at two incorporation levels; and (iv) to model polyphenol release kinetics over 42‐day refrigerated storage and identify the dominant release mechanism. To our knowledge, this is the first study to apply this clean‐label carrier system to *N. cadamba* bark extract in a probiotic yogurt system.

## 2. Materials and Methods

### 2.1. Chemicals, Reagents, and Plant Materials

All chemicals and reagents used in this study were of analytical grade, procured from Sigma Chemicals Co. (St. Louis, Missouri, United States). Food‐grade encapsulation carriers, namely *Quillaja* saponin and sunflower lecithin, were sourced from commercial suppliers as described by Gupta et al. [18]. Solvents employed for extraction and solubility profiling included distilled water, ethanol, dimethyl sulfoxide (DMSO), and chloroform [36]. Fresh *N. cadamba* bark was collected, authenticated, and cleaned prior to processing. The bark was shade‐dried, pulverized to a uniform powder using a mechanical grinder, and stored in airtight containers at ambient temperature until extraction.

### 2.2. Extraction of Bioactive Compounds

Extraction was performed using two solvent systems: 100% distilled water and a 1:1 hydroethanolic mixture (v/v), and two methodologies, AAE and UAE, to systematically evaluate the influence of solvent polarity and mechanical energy on phytochemical recovery. A fixed solid‐to‐solvent ratio of 1:20 (w/v) was used in all extractions. For AAE, the powdered bark was dispersed in the respective solvent and stirred using a high‐speed magnetic stirrer at 4000 rpm for 2 h at ambient temperature (25°C). For UAE, the bark powder was subjected to ultrasonication using an ultrasonic processor (model: UP400S, Hielscher Ultrasonics GmbH, Germany; tip diameter: 22 mm) at a constant output power of 90 W for 40 min under continuous agitation, utilizing the acoustic cavitation effect to disrupt cell walls and enhance mass transfer of bioactive compounds. Following extraction, all samples were centrifuged, filtered through Whatman No. 1 filter paper, and subjected to vacuum‐drying at 35°C for 10 h, followed by lyophilization at −40°C to yield a concentrated dry extract suitable for further characterization and encapsulation [7]. Solubility profiling of the lyophilized and vacuum‐concentrated extracts was subsequently conducted across a polarity gradient of organic solvents (distilled water, ethanol, DMSO, and chloroform) to determine the optimal extraction system for encapsulation. For solubility profiling, 10 mg of each dried extract was added to 1 mL of each solvent and equilibrated at 25°C for 60 min under continuous orbital agitation at 200 rpm. Samples were then centrifuged at 10,000 rpm for 10 min and the supernatant collected; solubility (%) was calculated as (dissolved mass/total mass) × 100, where dissolved mass was estimated by gravimetric determination of the supernatant residue after evaporation.

### 2.3. Microencapsulation by Spray‐Drying

**Figure 1:**
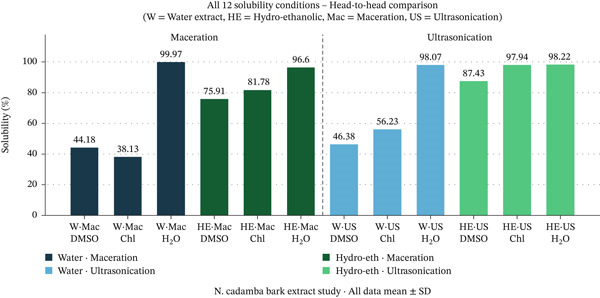
Comparative solubility profile of lyophilized versus vacuum‐concentrated *Neolamarckia cadamba* bark extracts in selected organic solvents.

The ultrasonicated hydroethanolic extract, which demonstrated the highest total phenolic content (TPC) and antioxidant activity (Figure 1), was selected for encapsulation. Spray‐drying was employed as the principal encapsulation methodology using a laboratory‐scale mini spray dryer (Büchi B‐290, Büchi Labortechnik AG, Switzerland) with the following optimized process parameters: inlet temperature of 160°C, outlet temperature of 80°C, aspirator speed of 1350 rpm, feed flow rate of 5 mL/min, and atomization air flow of 35 mm (nozzle diameter 0.7 mm). The feed emulsion was prepared at a total solids content of 10% (w/v) in distilled water. The wall material system comprised *Quillaja* saponin and sunflower lecithin at a 1:2 ratio (w/w). This combination was strategically selected for its complementary functionality: *Quillaja* saponin, a natural triterpenoid glycoside surfactant, provides amphiphilic interfacial stabilization and enhances polyphenol loading capacity, whereas sunflower lecithin, a phospholipid‐rich emulsifier, contributes to bilayer formation and gastric protection of the encapsulated bioactives. Encapsulation efficiency (EE%) was calculated as EE*%* = [(TPC_0_ − TPC_ _surface_)/TPC_0_] × 100, where TPC_0_ is the TPC of the feed emulsion and TPC_surface_ is the phenolic content of the surface (unencapsulated) fraction extracted by surface washing with distilled water prior to full solvent extraction. Prior to spray‐drying, the concentrated extract was homogenized with the wall material solution using a high‐speed homogenizer at 10,000 rpm for 3 min to form a stable feed emulsion, specifically targeting the sequestration of astringent tannins to prevent their premature interaction with the dairy matrix [13, 18]. The resulting encapsulated powder (encapsulated *N. cadamba* extract) was collected, stored in airtight amber vials at ambient temperature, and characterized for physical and chemical properties.

### 2.4. Physical Characterization of Encapsulated Particles

The colloidal stability of the optimized feed emulsion was assessed using DLS, which quantified the Z‐Average particle size and polydispersity index (PdI), providing insight into particle uniformity and nanodispersion quality. Electrostatic stability was evaluated through zeta potential analysis; values with absolute magnitudes exceeding |30| mV were considered indicative of strong interparticle repulsion and long‐term colloidal stability. Surface morphology of the spray‐dried powder was examined by SEM at 2000× magnification, enabling high‐resolution visualization of particle geometry, surface integrity, and the absence of fractures or agglomerations [36]. Elemental composition and purity were determined by EDX. The physical state of the encapsulated powder (crystalline versus amorphous) was assessed by XRD, and thermal stability was evaluated by TGA across a defined temperature gradient to simulate food processing conditions [16].

### 2.5. Molecular Characterization by FTIR Spectroscopy

FTIR spectroscopy was performed on both the spray‐dried encapsulated powder and the final fortified probiotic yogurt over the wavenumber range of 4000–400 cm^−1^ using an attenuated total reflectance (ATR) mode. This dual‐phase analysis served two purposes: (i) to confirm the chemical retention of key functional groups particularly O‐H stretching (3200–3500 cm^−1^) and C = O/C = C stretching (~ 1650 cm^−1^) indicative of MIAs and phenolic compounds within the wall matrix postprocessing; and (ii) to verify the molecular compatibility between the encapsulated extract and the complex dairy matrix in the fortified yogurt, by monitoring the stability of Amide I (1640–1700 cm^−1^) and Amide II (1510–1580 cm^−1^) protein bands as markers of structural integrity and the absence of undesirable chemical cross‐linking [6, 9].

### 2.6. Probiotic Yogurt Preparation and Formulation Design

Probiotic yogurt was prepared by inoculating standardized, heat‐treated (85°C for 15 min) cow′s milk with a commercial NCDC 144 starter culture containing *Lactobacillus delbrueckii* subsp. *bulgaricus* and *S. thermophilus* at a ratio of 1:1 (v/v) [11]. Formulations were designed to directly compare the effect of free extract (free *N. cadamba* extract) versus encapsulated extract (encapsulated *N. cadamba* extract) on the physicochemical and sensory attributes of probiotic yogurt. Both extract forms were incorporated at concentrations of 1% and 2% (w/v) into the inoculated milk prior to incubation at 42°C until the target pH of 4.5 was achieved. A plain yogurt without any extract addition served as the control. All formulations were prepared in triplicate and immediately transferred to refrigerated storage at 4°C following fermentation.

### 2.7. Antioxidant Activity and TPC

The antioxidant potency of the extracts and fortified yogurt formulations was quantified using three complementary radical scavenging assays: the DPPH (2,2‐diphenyl‐1‐picrylhydrazyl) assay, the ABTS (2,2 ^′^‐azino‐bis (3‐ethylbenzothiazoline‐6‐sulfonic acid)) assay, and the SOSA (superoxide anion scavenging activity) assay, with results expressed as percentage inhibition relative to a Trolox standard curve. TPC was determined using the Folin–Ciocalteu colorimetric method and expressed as milligrams of gallic acid equivalents per gram of dry extract (mg GAE/g). All assays were conducted in triplicate, and measurements were performed at defined time points throughout the 42‐day storage period to monitor retention of antioxidant activity in the yogurt matrix.

### 2.8. Release Kinetics of Encapsulated Polyphenols

The sustained release behavior of polyphenolic compounds from the spray‐dried encapsulated powder into the probiotic yogurt matrix was monitored over a 42‐day refrigerated storage period. At defined time intervals (Days 1, 7, 14, 21, 28, 35, and 42), samples were withdrawn, centrifuged, and the supernatant analyzed for released TPC using the Folin–Ciocalteu method. The cumulative percentage release was calculated relative to the TPC of the encapsulated powder determined prior to incorporation. Release data were fitted to established kinetic models including zero‐order, first‐order, Higuchi diffusion, Korsmeyer–Peppas, and Hixson–Crowell models to elucidate the dominant release mechanism governing polyphenol liberation from the saponin–lecithin wall matrix. The goodness of fit was assessed by the coefficient of determination (*R* ^2^) and the root mean square error (RMSE) for each model, with the best‐fitting model selected based on the highest *R* ^2^ value (≥ 0.98) and lowest RMSE [38, 39].

### 2.9. Texture Profile Analysis (TPA)

Textural characteristics of all yogurt formulations were quantified by TPA using a texture analyzer equipped with a cylindrical probe (36 mm diameter) at a test speed of 1 mm/s and 30% strain. A two‐cycle compression test was performed on chilled yogurt samples (4°C) to derive the following TPA parameters: Hardness (g), Adhesiveness (g·sec), Springiness (dimensionless), Cohesiveness (dimensionless), Gumminess (g), and Chewiness (g). These parameters collectively describe the rheological behavior of the yogurt gel matrix and serve as critical indicators of mouthfeel, structural integrity, and consumer acceptance in set‐type yogurt products [21].

### 2.10. Sensory Evaluation

Sensory evaluation was conducted by a panel of 30 semitrained evaluators (15 male, 15 female; aged 22–45 years) under standardized, controlled conditions. Formal ethical committee review was not required for this study because all test materials were prepared exclusively from food‐grade, edible ingredients (probiotic yogurt, *Quillaja* saponin, sunflower lecithin, and *N. cadamba* bark extract at concentrations used in traditional food preparation), and the sensory procedure involved only voluntary tasting of safe food products without any invasive procedures, deception, or collection of personal health data; this exemption is consistent with institutional policy for food sensory studies involving GRAS (generally recognized as safe) materials. All panelists were fully informed of the nature, purpose, and voluntary character of the study and provided written consent to participate prior to evaluation. Yogurt samples were assessed for color and appearance, aroma, flavor and taste, mouthfeel, texture, and overall acceptability using a 9‐point hedonic scale (1 = *dislike extremely*; 9 = *like extremely*). Samples were served in coded, randomized order at refrigerated temperature (4°C), with still water provided for palate neutralization between evaluations. Sensory data were analyzed to identify statistically significant differences in attribute scores between free extract and encapsulated formulations, with particular attention to flavor masking efficacy.

### 2.11. Statistical Analysis

All experiments were performed in triplicate and results expressed as mean ± standard deviation (SD). Data were subjected to one‐way analysis of variance (ANOVA) using SPSS (V26.0, IBM Corp., United States) and OriginPro software. Statistically significant differences between formulations were identified using Tukey′s Honestly Significant Difference (HSD) and for multiple variables, Duncan′s Multiple Range Test (DMRT) post hoc test at a significance level of *p* < 0.05 was applied. Prior to ANOVA, normality of data distribution was confirmed by the Shapiro–Wilk test, and homogeneity of variances was verified using Levene′s test. *p* values are reported in figure captions and tables throughout. Graphical representation of release kinetics, antioxidant profiles, and textural data was performed using OriginPro 2023.

## 3. Results and Discussion

### 3.1. Solubility Profile and Extraction Efficiency

The findings regarding the extraction efficiency of *N. cadamba* bark bioactives are consistent with established principles of phytochemistry, particularly the like‐dissolves‐like rule and the mechanisms of cavitation‐enhanced mass transfer [3, 5]. The near‐perfect solubility of the 100% distilled water extract (99.97 ± 0.06%) obtained via maceration indicates the prevalence of highly polar, water‐soluble compounds in the bark, aligning with traditional uses of aqueous extracts from medicinal plants [4]. For instance, polysaccharides from *N. cadamba* fruits, which are highly water‐soluble, have been successfully extracted using hot water methods [33]. The slightly lower but comparable (Figure 1) solubility (98.07 ± 0.16%) with ultrasonication in water suggests that although mechanical agitation aids, the primary compounds are readily accessible in an aqueous medium [41, 42]. Kindly Insert figure 1 here.

Figure 1 illustrates the relative solubility of *N. cadamba* bark extracts across a polarity gradient of organic solvents, comparing two distinct drying methodologies: lyophilization and vacuum concentration.

Conversely, the 1:1 hydroethanolic extract demonstrated superior affinity for organic solvents, achieving 97.94 ± 0.21% solubility in chloroform and 87.43 ± 2.13% in DMSO when ultrasonication was employed (Figure 1). This disparity strongly suggests that ultrasonication is crucial for the efficient liberation of moderately polar compounds, such as certain phenolic compounds and alkaloids, which are typically more soluble in hydroethanolic systems [4, 43]. The high‐energy acoustic cavitation generated by ultrasonication enhances cell disruption and mass transfer, thereby improving the extraction yield of these compounds compared with conventional methods [6, 9, 44]. Previous research has confirmed the effectiveness of UAE for enhancing the recovery of bioactive compounds from various plant matrices [41, 45, 46]. This solvent‐method synergy is critical for obtaining a comprehensive phytochemical profile, as different compounds exhibit varying solubilities and require distinct extraction conditions [3, 5, 44].

### 3.2. Antioxidant Potency and TPC

**Figure 2:**
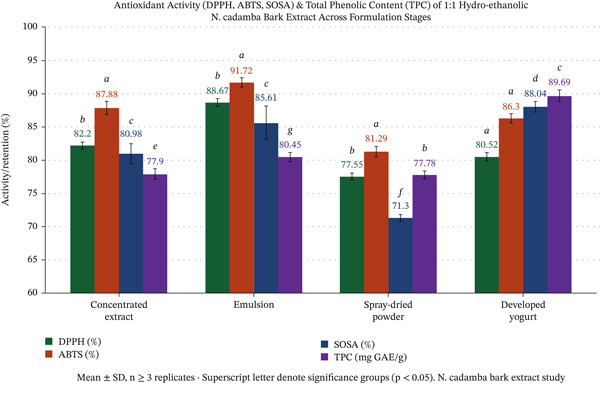
Comparative analysis of antioxidant capacities and total phenolic content (TPC) of *N. cadamba* bark extracts across the formulation stages.

The functional evaluation of the extracts through DPPH and ABTS assays clearly established the superior radical scavenging potential of the hydroethanolic system. The concentrated 1:1 hydroethanolic extract′s DPPH scavenging activity of 83.22 ± 0.35% and ABTS activity of 91.29 ± 0.93% significantly surpassed that of the pure water extract (Figure 2). This indicates that the hydroethanolic extraction method successfully recovered a higher concentration of potent antioxidant compounds from *N. cadamba* bark [4]. This is consistent with studies showing that hydroethanolic extracts of medicinal plants often possess higher antioxidant capacities due to their richer phenolic and flavonoid content compared to aqueous extracts [47, 48].

Figure 2 depicts the free radical scavenging potential and phenolic concentration of various *N. cadamba* bark extracts. The results evaluate the efficacy of the extraction methods in preserving the secondary metabolites essential for functional food applications.

Crucially, the preservation of antioxidant activity during the production of the probiotic yogurt further supports the efficacy of the hydroethanolic formulation. The final probiotic yogurt containing the encapsulated hydroethanolic extract retained a TPC of 71.47 ± 0.53 mg/g and a DPPH activity of 72.94 ± 4.53%. In contrast, although the water‐based probiotic yogurt maintained a respectable ABTS activity of 77.73 ± 0.99%, its DPPH scavenging ability significantly decreased to 15.11 ± 0.76% in the final product (Figure 2). This differential retention suggests that the phytochemicals recovered via the hydroethanolic method are more resilient to the acidic conditions and microbial metabolism encountered during lactic acid fermentation in probiotic yogurt [28, 49]. This resilience may be attributed to the enrichment of stable, nonglycosylated flavonoid aglycones (e.g., quercetin and gallic acid), which have been identified in *N. cadamba* extracts, and/or specific indole alkaloids that are less susceptible to degradation by the *Lactobacillus* strains commonly used in probiotic yogurts [50, 51]. Conversely, the sharp DPPH decline in the aqueous formulation implies the preferential extraction of labile, highly polar phenolic glycosides or hydroxycinnamic acids that are prone to enzymatic degradation by *β*‐glucosidases produced by certain probiotic bacteria [52].

It should be noted that the antimicrobial properties of *N. cadamba* extracts [8] may influence starter culture kinetics; however, viable counts of *L. delbrueckii* subsp. *bulgaricus* and *S. thermophilus* were not measured in the present study, which represents a significant limitation. Tannins at elevated concentrations are known to inhibit certain *Lactobacillus* strains [52]; however, encapsulation within the saponin–lecithin matrix is expected to prevent premature extract–culture contact during fermentation, thereby attenuating potential inhibitory effects, consistent with the findings of Rogalska et al. [19], who demonstrated that encapsulated cocoa phenolics had a neutral‐to‐mildly‐stimulatory effect on LAB. Future work should confirm this by monitoring acidification kinetics (pH drop rate) and enumerating viable counts at defined storage intervals (Days 0, 7, 14, 21, 28, 35, and 42) for control, free‐extract, and encapsulated‐extract yogurt groups.

### 3.3. Particle Stability and Structural Morphology

**Figure 3:**
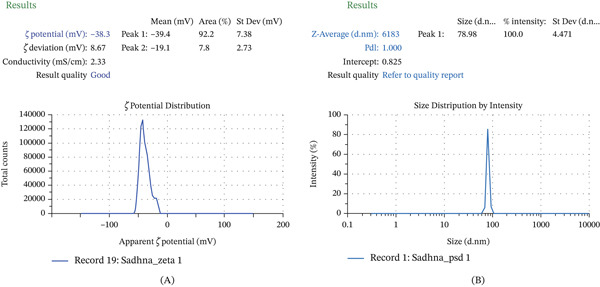
(A) Zeta potential distribution and (B) surface charge analysis of optimized *N. cadamba* extract emulsion.

The rigorous structural characterization and colloidal stability testing confirmed the success of the encapsulation process. The optimized emulsion exhibited a principal intensity peak diameter of 78.98 nm, representing the dominant nano‐sized population within the formulation (Figure 3). The cumulants Z‐average of 6183 nm reflect the presence of polydisperse aggregates in the feed emulsion prior to spray‐drying and should not be interpreted as the particle size of the final powder; the relevant nano‐sized fraction is characterized by the intensity peak at 78.98 nm. This physical stability is further corroborated by a zeta potential of −38.3 mV [53]. Values with magnitudes greater than 30 mV typically indicate excellent electrostatic repulsion between particles, which effectively prevents aggregation or sedimentation during storage, thus ensuring long‐term colloidal stability [54].

Figure 3 illustrates the electrokinetic potential (zeta potential) of the optimized nano‐emulsion encapsulated with *N. cadamba* bark extract. This measurement serves as a critical indicator of the physical stability and storage life of the colloidal system.

**Figure 4:**
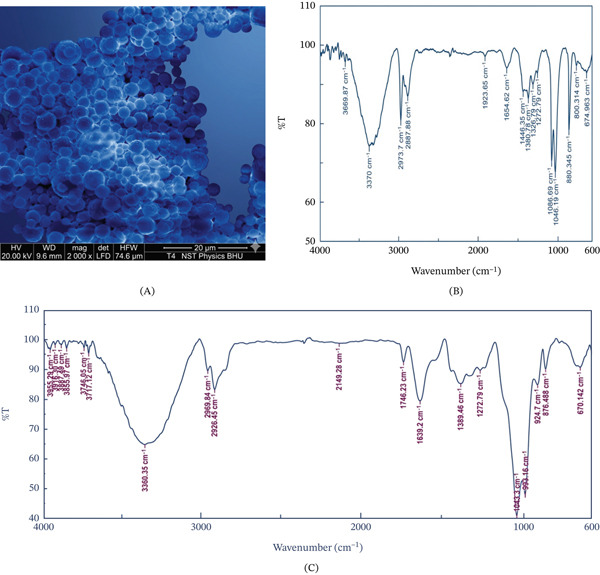
Characterization of encapsulated *N. cadamba* bark extract: (A) SEM surface morphology; (B) FT‐IR spectroscopic profile; and (C) matrix compatibility (FT‐IR probiotic yogurt).

Morphological observations via SEM of the encapsulated powder revealed that spherical, fracture‐free encapsulated particles are essential for maintaining a continuous protective barrier around the bioactive compounds (Figure 4A). This integrity is critical for controlled release and protection against environmental stressors [55].

Figure 4 illustrates the chemical and physical properties of the optimized *N. cadamba* bark extract powder. (A) Surface morphology (SEM): The micrographs of the spray‐dried powder reveal the successful fabrication of microcapsules. The particles exhibit a predominantly spherical geometry with characteristic surface indentations and a continuous, crack‐free “crust.” This intact structural integrity serves as a physical barrier against environmental stressors, providing a physical barrier against oxidative degradation and thermal damage during storage. (B) Molecular fingerprinting (FT‐IR powder): The infrared spectra of the dry encapsulated powder confirm the chemical retention of key secondary metabolites. The broad peak at 3200–3500 cm^−1^ (O‐H stretching) and the sharp absorption near 1650 cm^−1^ (C = O/C = C stretching) indicate that the MIAs and phenolic compounds remain structurally intact within the wall matrix after processing. (C) Matrix compatibility (FT‐IR probiotic yogurt): The spectra of the fortified probiotic yogurt demonstrate the successful integration of the nano‐encapsulated extract into a complex food system. The stability of the Amide I and II peaks (1640–1545 cm^−1^) of the yogurt proteins, combined with the persistence of characteristic bioactive vibrational bands, is consistent with reduced undesirable chemical cross‐linking between the dairy matrix and the encapsulated bioactives, and with the maintained structural integrity of the wall matrix in a high‐moisture environment.

The chemical identity of the entrapped bioactives was confirmed using FTIR spectroscopy, with data consistent with the core extract (Figure 4B,C) is consistent with integration within the *Quillaja* saponin and sunflower lecithin wall materials and probiotic yogurt without adverse chemical degradation, as inferred from the stability of Amide I (1640–1700 cm^−1^) and Amide II (1510–1580 cm^−1^) protein bands in the encapsulated‐extract yogurt (Figure 4C), which suggests reduced protein–polyphenol crosslinking relative to the free‐extract yogurt. The elevated hardness (230.05 ± 0.052 g) and chewiness (178.51 ± 0.38 g) of the free‐extract formulation are consistent with tannin–casein interaction driving gel densification [4, 56]. It should be noted that the nano‐shield mechanism is presented as a hypothesis‐consistent inference drawn from TPA hardness reduction and FTIR Amide band stability; molecular‐level confirmation (e.g., NMR, isothermal titration calorimetry) was not performed and is recommended for future work [6, 9, 57]. This synergistic wall system, combining a natural triterpenoid surfactant like *Quillaja* saponin with sunflower lecithin, is known to enhance polyphenol loading and gastric protection, representing an advanced strategy in encapsulation technology. Such all‐natural protein‐polysaccharide or lipid‐based conjugates have shown great promise in stabilizing bioactive compounds and improving their delivery [57].

### 3.4. TPA and Rheological Impact

**Figure 5:**
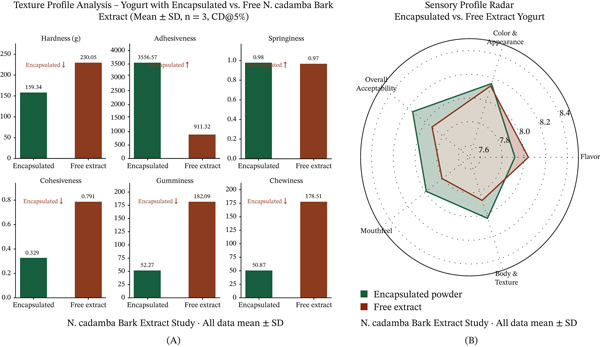
(A) Integrated textural and (B) sensorial profile of probiotic yogurt fortified with *N. cadamba* bark extract.

The incorporation of both free and encapsulated extracts notably influenced the rheological properties of the probiotic yogurt gel matrix. TPA showed that probiotic yogurt fortified with free extract (Figure 5, left) exhibited the highest hardness (230.05 ± 0.052 g) and chewiness (178.51 ± 0.38 g). This increase in hardness is potentially due to direct interactions between tannins, which are abundant in *N. cadamba*, and milk proteins like casein, leading to cross‐linking and a denser gel network [4, 56].

Figure 5 presents a multidimensional evaluation of the final food product, correlating the mechanical properties of the yogurt gel with human organoleptic perception to compare the performance of free versus encapsulated *N. cadamba* extract formulations (all TPA data: mean ± SD, *n* = 3; all sensory data: mean ± SD, *n* = 30 per treatment, assessed on a 9‐point hedonic scale). (A) TPA: Fortification with encapsulated extract significantly optimized the yogurt structure relative to the free‐extract formulation, yielding lower Hardness (159.34 ± 0.12 g vs. 230.05 ± 0.052 g; *p* < 0.0001), higher Adhesiveness (3556.57 ± 0.26 g·s vs. 911.32 ± 0.24 g·s; *p* < 0.0001), and reduced Gumminess (52.27 ± 0.21 g vs. 182.09 ± 0.089 g; *p* < 0.0001) and Chewiness (50.87 ± 0.49 g vs. 178.51 ± 0.38 g; *p* < 0.0001), collectively confirming a smoother mouthfeel and reduced polyphenol–protein cross‐linking. Cohesiveness also differed significantly (*p* < 0.0001), whereas Springiness (0.98 ± 0.0045) was the sole parameter that did not differ significantly between formulations (*p* = 0.055), indicating preserved elastic recovery. (B) Sensory evaluation: No statistically significant differences were observed between encapsulated and free *N. cadamba* extract for any sensory attribute—Color and Appearance (8.15 ± 0.32 vs. 8.13 ± 0.29; *p* = 0.638), Flavor (*p* = 0.743), Body and Texture (p = 0.913), Mouthfeel (*p* = 0.832), or Overall Acceptability (8.16 ± 0.36 vs. 7.92 ± 0.27; *p* = 0.949) indicating that encapsulation delivered superior textural performance without adversely affecting organoleptic acceptability.

In contrast, the formulation containing encapsulated powder displayed the maximum adhesiveness (3556.57 ± 0.26 g·s) compared with the free‐extract formulation (911.32 ± 0.24 g·s; *p* < 0.0001). This suggests that the lipid‐based wall materials, particularly lecithin, contribute to a smoother, more cohesive mouthfeel by influencing the lipid–protein interactions within the yogurt matrix [51]. These variations indicate that although free extracts might over‐harden the gel, microencapsulation offers a viable strategy for fortifying probiotic yogurt without compromising its characteristic creamy texture. Encapsulation effectively acts as a structural modifier, enhancing consumer‐relevant parameters like springiness and adhesiveness (Figure 5, right), which are critical for consumer acceptance of functional food products [58]. The careful selection of encapsulation materials can thus mitigate negative textural impacts while delivering beneficial compounds [36].

### 3.5. Release Kinetics and Sustained Delivery

**Figure 6:**
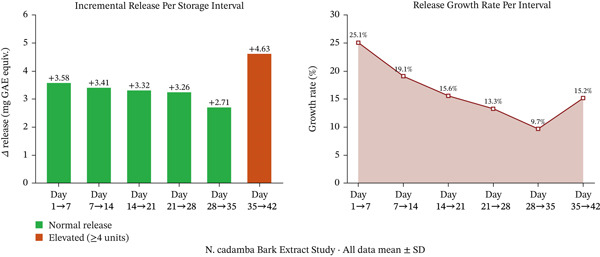
Release kinetics and incremental growth assessment of encapsulated *N. cadamba* polyphenolics.

The long‐term functional efficacy of the spray‐dried microencapsulated powder was thoroughly evaluated through a 42‐day release kinetics (Figure 6) study under refrigerated conditions. The results demonstrated a controlled and gradual diffusion of polyphenolic compounds into the probiotic yogurt matrix, initiating at 14.27 ± 0.02% on Day 1 and steadily increasing to 35.18 ± 0.03% by the end of the observation period. Mathematical modeling of the cumulative release data revealed that the zero‐order model provided the best fit (*R* ^2^ = 0.9967, RMSE = 0.3886; k_0_ = 0.4905 mg GAE/g/day), followed by the Hixson–Crowell model (*R* ^2^ = 0.9878, RMSE = 0.0304; k^H^ = 0.019853 day^−1^), the first‐order model (*R* ^2^ = 0.9771, RMSE = 0.0442; k_0_ = 0.02093 day^−1^), the Higuchi model (*R* ^2^ = 0.9594, RMSE = 1.3650; K^h^ = 3.7140 mg GAE/g·day^−½^), and the Korsmeyer–Peppas model (*R* ^2^ = 0.8953, RMSE = 0.0945; *n* = 0.2297). The superior fit of the zero‐order model (*R* ^2^ = 0.9967) indicates a near‐constant rate of polyphenol release from the saponin–lecithin wall matrix throughout refrigerated storage. The Korsmeyer–Peppas exponent *n* = 0.2297 (≤ 0.45) independently confirms that the underlying transport mechanism is Fickian diffusion (Case I transport), whereby release rate is governed by concentration gradient across the intact wall matrix rather than matrix erosion or swelling. These kinetic parameters are summarized in Table 1. This sustained release profile is characteristic of a well‐formed matrix‐type encapsulation system [38, 39].

**Table 1:** Release kinetics model parameters for encapsulated *N. cadamba* polyphenols in probiotic yogurt over 42 days (*n* = 3, mean ± SD).

Kinetic model	Rate constant	*R* ^2^	RMSE	Interpretation
Zero‐order (best fit)	K_0_ = 0.4905 mg GAE/g/day	**0.9967**	0.3886	Constant‐rate (zero‐order) release; best fit
First‐order	k_1_ = 0.02093 day^−1^	0.9771	0.0442	Concentration‐dependent release
Higuchi	k_0_ = 3.7140 mg GAE/g·day^−½^	0.9594	1.3650	Matrix diffusion; Fickian mechanism confirmed by K–P *n* = 0.2297
Korsmeyer–Peppas	*n* = 0.2297	0.8953	0.0945	Fickian diffusion (*n* ≤ 0.45); diffusion‐controlled transport confirmed
Hixson–Crowell	K^HC^ = 0.019853 day^−1^	0.9878	0.0304	Surface area reduction; erosion‐based release component

*Note:* K_0_, zero‐order rate constant (best fit); k_1_, first‐order rate constant; k_0_, zero‐order rate constant; *n*, Korsmeyer–Peppas release exponent; k^Hc^, Hixson–Crowell rate constant; bold *R* ^2^ value indicates best‐fit model selected on criteria *R* ^2^ ≥ 0.98 and lowest RMSE.

Abbreviation: RMSE, root mean square error.

This controlled mechanism ensures that the antioxidant benefits of *N. cadamba* are not rapidly depleted during the initial stages of storage but are instead delivered consistently throughout the product′s entire shelf life. Such a sustained release is crucial for providing a reliable source of functional nutrition to the consumer over time [59, 60]. The diffusion‐controlled kinetics observed are typical for amphiphilic biopolymer wall systems like those involving saponin–lecithin, which facilitate a gradual release of encapsulated cargo [57]. This approach represents a significant step toward ensuring the stability and efficacy of functional ingredients in food products over extended storage periods.

Figure 6 details the release profile of encapsulated polyphenols from the *N. cadamba* bark extract powder over a 42‐day storage period. The data quantify the controlled liberation of polyphenolic compounds from the spray‐dried wall matrix into the probiotic yogurt. During the initial 5 weeks (Days 1–35), the system maintained a consistent release rate, with incremental increases ranging from +3.58 to +2.71 mg GAE/g, supporting the efficacy of the saponin–lecithin matrix in preventing burst release. The release increment declined gradually from 25.1% in the first week to 9.7% by the fifth week, consistent with Fickian matrix diffusion in which the diffusion path lengthens as outer encapsulant layers are depleted. A surge in incremental release (+4.63 mg GAE/g; 15.2%) was observed in the final interval (Days 35–42), possibly reflecting structural loosening of the matrix at the end of the storage cycle. Throughout the majority of the storage period, the encapsulation system maintained a low and steady release rate, consistent with controlled polyphenol diffusion and preserved antioxidant activity over refrigerated storage.

### 3.6. Comprehensive Physicochemical Profiling (EDX, TGA, and XRD)

**Figure 7:**
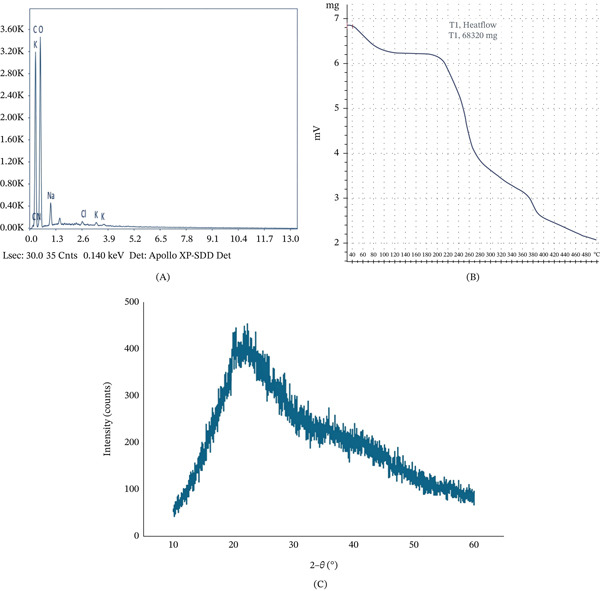
Physicochemical characterization of spray‐dried *N. cadamba* microcapsules: (A) elemental composition (EDX), (B) thermal stability (TGA), and (C) crystallographic profile (XRD).

The microstructural and thermal properties of spray‐dried *N. cadamba* bark extract powder were characterized to evaluate the efficiency of the encapsulation process. The combined results from EDX, TGA, and XRD confirm that the optimized formulation possesses the requisite purity and stability for food applications (Figure 7). Nanotechnology significantly impacts the delivery of phytobioactive compounds, addressing issues like low aqueous solubility, poor biostability, and lack of target specificity, which are often characterized by these analytical methods [61]. The EDX spectra revealed a high‐purity elemental profile, dominated by carbon (C) (52.4%) and O (44.1%), with trace amounts of nitrogen and minerals indigenous to the bark. The absence of heavy metal peaks confirms that the ultrasound‐assisted aqueous extraction effectively isolated the organic bioactives without introducing inorganic contaminants. This confirms the safety of the extract for human consumption within a probiotic yogurt matrix. The focus on natural, plant‐derived ingredients in nanocarriers aligns with the goal of reducing toxicity and ensuring product safety [62, 63]. The selection criteria for nanomaterials heavily emphasize biocompatibility and safety, which direct the avoidance of toxic components and favor GRAS materials for food applications [61, 64]. TGA demonstrated that the encapsulated powder maintains excellent thermal integrity across a wide temperature range. An initial weight loss of approximately 6.2% was observed up to 110°C, corresponding to the loss of adsorbed moisture. The major thermal degradation phase occurred between 220°C and 380°C, involving the depolymerization of the wall material and the decomposition of MIAs. The stability of the extract up to 210°C indicates that it can easily withstand the thermal rigors of yogurt pasteurization and industrial spray‐drying. Encapsulation by nanocarriers is recognized for significantly improving the stability of bioactive compounds against environmental factors like heat [65, 66]. For instance, essential oils, which are highly susceptible to thermal degradation, show enhanced stability when encapsulated within mesoporous silica nanoparticle. This enhanced stability directly addresses challenges faced by many lipophilic compounds during food processing [66, 67]. The XRD patterns of the encapsulated samples displayed a broad, diffuse amorphous halo with a complete lack of sharp crystalline reflections. This confirms that the *N. cadamba* bioactives were successfully molecularly dispersed within the wall matrix during the rapid drying process. This amorphous state is a critical finding, as it facilitates rapid dissolution and enhances the solubility of the otherwise hydrophobic bark constituents. Overcoming the low aqueous solubility and poor bioavailability of phytochemicals is a key advantage of nanotechnology [68, 69]. By converting crystalline structures into an amorphous state, nanocarriers can significantly improve the dissolution rate and subsequent absorption of poorly soluble compounds [61]. The synergy between these three analyses provides a robust validation of the encapsulation strategy. The EDX confirms that the extraction remains a clean, food‐grade process, aligning with the principles of green synthesis and safe application of herbal extracts [70]. The TGA data demonstrate that the encapsulated powder maintained thermal stability up to 210°C, consistent with protection of the bioactives from thermal degradation, a well‐documented benefit of nanoencapsulation for sensitive compounds like essential oils [66, 67]. The XRD results suggest a mechanistic basis for potentially improved dissolution kinetics; converting the extract from a crystalline raw state into an amorphous dispersion is expected to favor faster solubilization and availability of bioactives under gastrointestinal conditions, though direct in vitro digestion studies are required to confirm bioaccessibility gains [68, 69]. This integrated approach illustrates how nanoencapsulation strategies can address inherent physicochemical limitations of herbal compounds, improving their formulation stability and controlled release performance within food matrices [71, 72].

Figure 7 provides a comprehensive analysis of the encapsulated *N. cadamba* bark extract, evaluating its elemental purity, thermal decomposition behavior, and physical phase state. (A) Elemental analysis (EDX): The EDX spectra confirm the primary elemental composition of the microcapsules, predominantly consisting of C and O from the carbohydrate wall materials and organic bioactives. The absence of heavy metal peaks or unexpected mineral contaminants confirms the high purity of the “pure water” extraction and the safety of the encapsulation process for functional food use. (B) Thermal stability (TGA): The thermogravimetric curves illustrate the multistage weight loss profile of the powder. An initial minor weight loss (100°C) is attributed to moisture evaporation, followed by a robust stability region. This confirms that encapsulation successfully protects the *N. cadamba* bioactives from the high temperatures typically used in industrial food processing. (C) Physical state analysis (XRD): The XRD pattern reveals the crystallographic nature of the powder. The appearance of a broad, “hump‐like” peak (amorphous halo) and the absence of sharp crystalline reflections indicate that the *N. cadamba* extract is dispersed within the wall matrix in an amorphous state. This amorphous structure is highly desirable in food engineering because amorphous dispersions are known to exhibit enhanced aqueous solubility and faster dissolution rates compared to crystalline forms, which is expected to facilitate polyphenol release into the food matrix and favor gastrointestinal availability in future digestion studies [68, 69].

## 4. Conclusion

This research successfully demonstrates the potential of *N. cadamba* bark extract as a high‐value functional additive in probiotic yogurt. The study confirms that the efficacy of the final product is deeply rooted in the synergy between advanced extraction techniques and robust encapsulation strategies. Ultrasonication emerged as a superior method for liberating bioactives through acoustic cavitation, whereas spray drying provided a necessary protective shield, preventing the degradation of antioxidants in the acidic dairy environment. The structural and rheological analysis showed that although fortification alters the probiotic yogurt′s gel network, the use of encapsulated powder yields a more favorable texture characterized by higher adhesiveness and smoothness compared to free extracts. Most importantly, the findings are consistent with the conclusion that the inherent sensory acceptability of the encapsulated formulation was comparable to the free‐extract formulation across all attributes (all *p* > 0.05), indicating that encapsulation did not adversely affect organoleptic properties while delivering superior textural performance. The controlled release profile over 42 days further validates the matrix stability of the system. Although this study establishes a strong physicochemical and sensory foundation for *N. cadamba* fortification, several avenues for future research remain to fully realize its commercial and health potential. Several limitations of the current study must be acknowledged. First, no in vitro digestion model (e.g., INFOGEST protocol) or Caco‐2 permeability assay was employed; consequently, the bioaccessibility and epithelial transport of the encapsulated polyphenols under gastrointestinal conditions remain unquantified. Second, probiotic viability counts (log CFU/g) were not measured during the 42‐day storage period, and therefore no conclusions can be drawn regarding the effect of the extract or the encapsulant on the survival of *L. delbrueckii* subsp. *bulgaricus* and *S. thermophilus*. Third, cytotoxicity of the *N. cadamba* bark extract on normal human cell lines was not assessed, which limits the available safety data to the EDX elemental purity profile. Fourth, the sensory panel (*n* = 30) was semitrained rather than expert, and no external validation of flavor masking has been conducted; it is noted that formal ethical committee approval was not required because all test materials were food‐grade edible ingredients and the evaluation involved only voluntary tasting, consistent with institutional exemption policy for GRAS food sensory studies. Fifth, scale‐up from laboratory spray‐dryer to pilot‐plant production has not been evaluated. These limitations should be addressed in future work before regulatory or commercial claims are made. Future studies should focus on in vivo bioavailability using animal models or simulated human gastrointestinal digestion trials to determine the actual metabolic pathways of the encapsulated polyphenols once consumed. Additionally, investigating how the bark extract influences the survival and growth of specific probiotic strains like *Lactobacillus* and *Bifidobacterium* during long‐term storage could illuminate potential prebiotic‐like effects. Transition from laboratory‐scale spray drying to pilot‐plant production will also be necessary to evaluate cost‐effectiveness and yield consistency. Finally, investigation of the molecular interactions between these encapsulated bioactives and the human gut microbiota, alongside in vivo pharmacokinetic studies, would provide the evidence base required to substantiate any future health claims associated with this functional yogurt.

## Author Contributions

Sadhna Mishra: conceptualization, methodology, investigation, and writing—original draft. Arvind Kumar: supervision, resources, and writing—review and editing. Vinay Kumar: formal analysis and data curation. Ashutosh Rai: investigation and resources. Manju Yadav: visualization and formal analysis. Vigya Mishra: methodology and investigation. Sunil Kumar Yadav: software and statistical analysis. Dipendra Kumar Mahato: supervision, conceptualization, writing—review and editing, and funding acquisition.

## Funding

No funding was received for this manuscript. Open access publishing facilitated by Deakin University, as part of the Wiley ‐ Deakin University agreement via the Council of Australasian University Librarians.

## Conflicts of Interest

The authors declare no conflicts of interest.

## Data Availability

The data that support the findings of this study are available from the corresponding author upon reasonable request.
